# Supplementary material for: The Effect of Cholesterol in MCF7 Human Breast Cancer Cells
Source: Int J Mol Sci. 2023 Mar 21;24(6):5935. doi: 10.3390/ijms24065935 (PMC10052157; doi:10.3390/ijms24065935)
Supplement: Supplementary file 1 [file ijms-24-05935-s001.zip › ijms-2285983-supplementary.pdf]

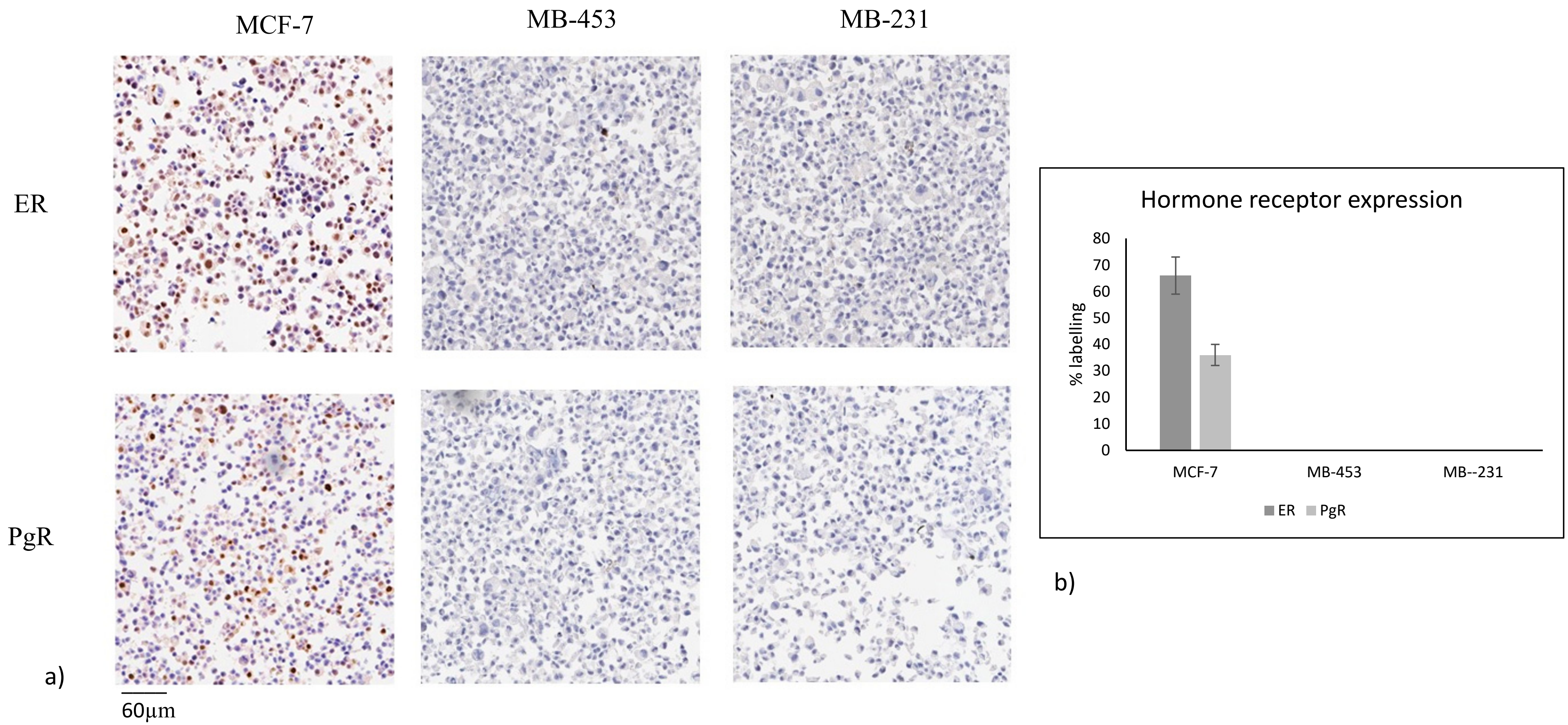

Figure S1. Characterization of cell lines. a)Immunohistochemical analysis of ER and PgR in MCF-7, MB-453 and MB-231; b) analysis of positive cells expressed in percent. Data are expressed as the mean ± S.D. of 2 independent experiments performed in duplicate. $* \ p < 0.001$

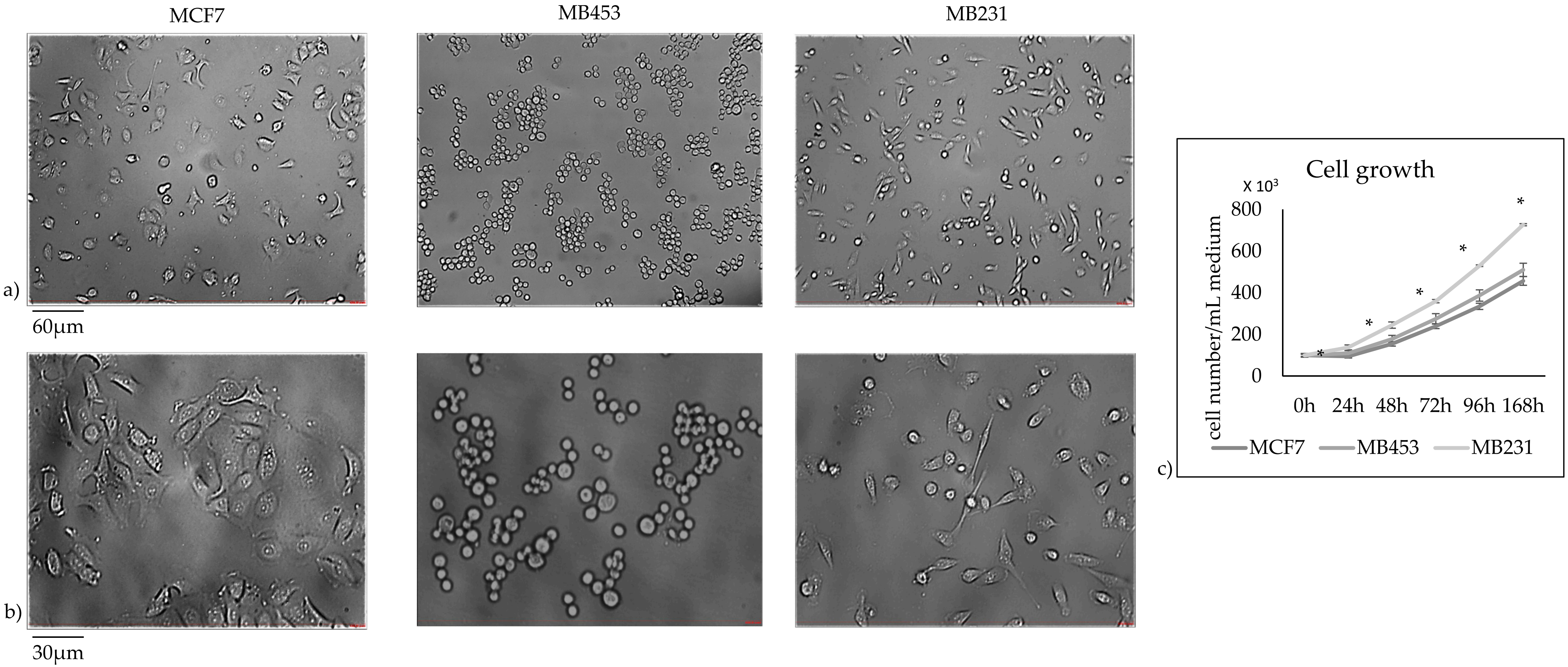

Figure S2. Characteristics of MCF7, MB453, MB231 cells after 48hrs of culture. a) cell morphology (20x magnification); b) cell morphology (40x magnification); c) cell growth at different times of cell culture. The cells were seeded at 100×10^3 cell concentration at 0h. Data are expressed as the mean ± S.D. of 3 independent experiments performed in duplicate. $* \; p < 0.05$ versus 0h.
